# Ultrasound-guided percutaneous versus trans-nasal pterygopalatine fossa block in endoscopic trans-sphenoidal pituitary gland surgery: a randomized controlled trial

**DOI:** 10.1186/s12871-026-03618-0

**Published:** 2026-02-02

**Authors:** Donia Hany Saad, Aly Mahmoud Moustafa Ahmed, Wael Medhat ElKholy, Mohamed Mohamed Bakr

**Affiliations:** 1 Department of Anaesthesia and Surgical Intensive Care, Faculty of Medicine, Kafrelsheikh University, Kafr El-Shaikh, Egypt; 2 Anaesthesia and Surgical Intensive Care, Faculty of Medicine, Alexandria University, Alexandria, Egypt; 3 Department of Neurosurgery, General Organization of Teaching Hospital and Institutes, Damanhour Medical National Institute, El Beheira, Egypt

**Keywords:** Pterygopalatine fossa block, Haemodynamic control, Bloodless surgical field, Endoscopic pituitary surgery, Ultrasound-guided anaesthesia.

## Abstract

**Background:**

Various techniques are being used to create a relatively bloodless surgical field during endoscopic trans-sphenoidal pituitary gland surgery (ETS). Pterygopalatine fossa (PPF) block helps to improve the field of surgery and control haemodynamic fluctuations.

**Methods:**

Eligible patients with the American Society of Anaesthesiologists class I or II were randomly classified by closed envelope method. Group TN received bilateral transnasal PPFB with 4 mL of 0.25% bupivacaine after induction of general anaesthesia. Group PC received bilateral ultrasound-guided infrazygomatic percutaneous PPFB with the same dosage and timing.

**Results:**

A total of 60 patients were included and randomized into the two groups. Between the two groups, there was no statistically significant difference in mean arterial blood pressure (MABP) and heart rate (HR). Fentanyl and dexametomidine were less significantly used intraoperatively in the PC group. Total intraoperative isoflurane consumption and the mean consumption per minute used intraoperatively were significantly higher in TN group (*p* < 0.001). Regarding Boezaart surgical field grading was no statistically significant difference between Group TN and Group PC at any time point.

**Conclusion:**

Ultrasound-guided percutaneous PPF block offers superior efficacy in reducing intraoperative anaesthetic consumption and opioid use compared to the transnasal approach in endoscopic transsphenoidal pituitary surgery. This technique contributes to enhanced haemodynamic stability and a more efficient anaesthetic management profile during surgery.

**Trial registration:**

Clinicaltrials.gov. Identifier NCT06836583, 24 February 2025.

**Supplementary Information:**

The online version contains supplementary material available at 10.1186/s12871-026-03618-0.

## Background

Endoscopic endonasal transsphenoidal resection of the pituitary gland is currently the most widely used method for removing pituitary tumor.The main goal in endoscopic pituitary gland surgery are minimal tissue manipulation and clear bloodless field with better panoramic visualization under haemodynamic stability and good postoperative analgesia to improve the outcome. often requiring effective perioperative analgesia [1]. 

The pterygopalatine ganglion (PPG), located within the pterygopalatine fossa (PPF), plays a crucial role in craniofacial pain transmission. It is connected to the maxillary nerve, parasympathetic fibers, and sympathetic pathways, making it an ideal target for regional anaesthesia in nasal, sinus, and skull base surgeries. PPF block is a regional analgesic technique that can enhance the operative field during ETS by maintaining haemodynamic stability and reducing intraoperative anaesthetic requirements [2–5]. 

The PPF block can be performed via transnasal application of local anaesthetic, transoral injection through the greater palatine foramen, or a percutaneous approach. Ultrasound (US) guidance in the percutaneous approach allows real-time visualization of soft tissues, surrounding vasculature, and bony structures within the PPF. It facilitates precise needle positioning and accurate anaesthetic deposition, confirming effective local anaesthetic spread. Therefore, US-guided percutaneous PPF block may offer a more successful blockade of the PPF contents compared to the transnasal approach [6]. 

In this study, we primarily aimed to evaluate how bilateral US-guided percutaneous PPF block compares to the transnasal approach on intraoperative anaesthetic requirements, guided by entropy monitoring used to maintain anaesthetic depth within a target range of 40–60. We also explored several clinically relevant outcomes, including total intraoperative dexmedetomidine and fentanyl usage, haemodynamic stability, surgical field quality, recovery pattern, and any associated side effects in conjugation with general anesthesia.

## Methods

This prospective, randomized, and quadruple blinded study was reviewed and approved by the institutional ethics committee in December 2024 (IRB number: KFSIRB200-494), and written informed consent was obtained from all participants. The trial adhered to the principles of the Declaration of Helsinki and was registered prior to patient enrollment at ClinicalTrials.gov (NCT06836583). This study also adhered to CONSORT guidelines. Based on a preliminary study by Mostafa et al. [7], a minimum sample size of 50 participants (25 per group) was required to detect a medium effect size (W = 0.4) in comparing the impact of bilateral ultrasound-guided percutaneous versus transnasal pterygopalatine fossa blocks on intraoperative anaesthetic requirements (as guided by entropy monitoring) during endoscopic transsphenoidal resection of pituitary adenoma under general anaesthesia. This sample size was calculated to achieve 80% statistical power at a 0.05 significance level using a Chi-square test with 1 degree of freedom, utilizing NCSS 2004 and PASS 2000 software [8].

The study was conducted between February 2025 and April 2025. Eligible patients were classified as American Society of Anaesthesiologists (ASA) physical status I or II, who were scheduled for endoscopic transsphenoidal pituitary gland surgery at two tertiary care centers [9]. Patients were randomly assigned to two equal groups using a closed-envelope method with concealed allocation. The study was conducted in a quadruple-blinded fashion. Patients with history of allergy or contraindication to any of the studied drugs, patients for whom pterygopalatine fossa block was contraindicated (patient refusal, fascial anomalies, coagulation disorder, skin infection at the injection site), obese patients with BMI more than 35 Kg/m^2^ were excluded. During the preoperative visit, all patients were informed with the procedure of PPFB either the percutaneous or trans nasal approach. They were instructed to fast overnight. After informed consent, the allocated patients were randomly assigned to one of two equal groups: Group TN: patients received bilateral trans-nasal PPFB using 4 ml 0.25% bupivacaine after the induction of GA. Group PC: patients in this group received bilateral ultrasound-guided infrazygomatic percutaneous PPF block with 4 mL of 0.25% bupivacaine on each side following the induction of general anaesthesia. Upon admission to the operating room, standard monitoring was applied, including electrocardiography, non-invasive blood pressure, core temperature via probe, entropy, capnography, pulse oximetry, end-tidal carbon dioxide (EtCO₂), and end-tidal anaesthetic agent concentration, measured using a GE Datex-Ohmeda anaesthetic gas analyzer. Anaesthesia induction was achieved with intravenous administration of propofol (1–2 mg/kg), fentanyl (1 µg/kg), and rocuronium bromide (0.6 mg/kg).

Ventilation was maintained using Datex-Ohmeda ventilator starting with oxygen flow 4 L/min then after 15 min flow was decreased to 1 L/min till the end of the surgery. End tidal carbon dioxide (ETCO_2_) was maintained at (30–35) mmHg. Oral packing was applied with direct laryngoscopy and Magill forceps, Intranasal xylometazoline was administered intranasal. After intubation, a bilateral US-guided PPF block was given to Group PC, patients were placed in the lateral head position.

**Fig. 1:**
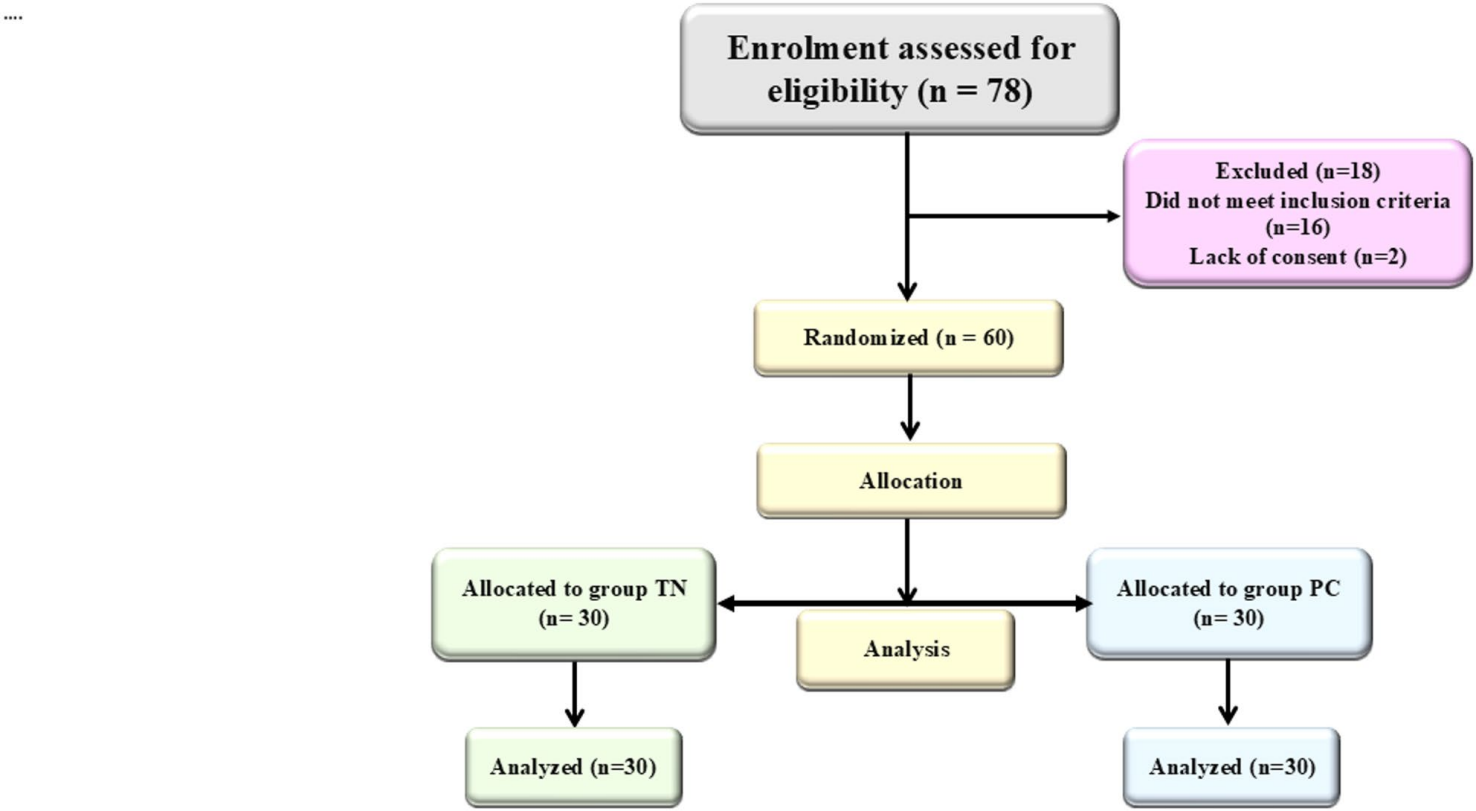
Flow chart showing the enrolment, allocation and analysis of the study participants

Following standard sterile preparation, a high-frequency linear ultrasound probe (Sonoscape model E1EXP) was placed horizontally on the lateral aspect of the face, just inferior to the zygomatic arch, superior to the mandibular notch, and anterior to the mandibular condyle (Fig. 1). This positioning enabled visualization of the maxillary bone, coronoid process of the mandible, lateral pterygoid muscle, lateral pterygoid plate, and the maxillary artery as it entered the pterygopalatine fossa. Using an in-plane technique, the needle was introduced parallel to the transducer and advanced from anterior to posterior toward the fossa. After confirming negative aspiration, 4 mL of 0.25% bupivacaine was administered deep to the lateral pterygoid muscle and plate.

In Group TN, patients were positioned in a 15-degree reverse Trendelenburg orientation, and the block was administered via the trans nasal approach between the middle and inferior turbinate. This passage was sterilized by a cotton-tipped applicator soaked with iodine solution. A 20-gauge/5-inch spinal needle was used after bending 2–3 mm of its tip along the port side with a sterile needle holder to form a 45 angle. The needle was lubricated with 5% lidocaine jelly, inserted into the nasal meatus and advanced with the bevel pointer facing laterally. Under endoscopic control (0_ optics, 4 mm diameter), the needle was inserted between middle and inferior turbinate. A total of 4 ml 0.25% bupivacaine was injected after negative aspiration just behind and over middle turbinate tail, where the pterygopalatine fossa was deeply located.

Anaesthesia was maintained using inhalational agents, with concentrations titrated to maintain entropy values between 40 and 60. Neuromuscular blockade was sustained with intermittent boluses of rocuronium (0.1 mg/kg), guided by peripheral nerve stimulation. Mechanical ventilation was adjusted to maintain end-tidal carbon dioxide (EtCO₂) levels within 35–40 mmHg. Intraoperative fluid management followed standardized anaesthesia fluid replacement guidelines [10]. All patients received intravenous paracetamol (15 mg/kg) administered over 15 min, in addition to dexamethasone (0.2 mg/kg, maximum 16 mg) and ondansetron (4 mg) for postoperative nausea and vomiting prophylaxis.

To optimize surgical field conditions, haemodynamic parameters were controlled with a target mean arterial pressure of approximately 65 mmHg. Pharmacologic management was implemented in a stepwise manner: initially, fentanyl (0.5 µg/kg) was administered to increase analgesia; if no response was observed within 5 min, a dexmedetomidine infusion was initiated, beginning with a loading dose of 0.5 µg/kg over 10 min followed by a maintenance infusion at 0.2–0.5 µg/kg/h.

Episodes of hypotension in both groups were treated by stopping dexmedetomidine, fluid challenge by isotonic saline, and or IV bolus of 5–10 mg ephedrine. Bradycardia, defined as a heart rate below 50 beats per minute [11], was managed with intravenous atropine at a dose of 0.01 mg/kg.

At the end of surgery, neuromuscular blockade was reversed with injection of neostigmine (0.04 mg/kg) with atropine (0.01 mg/kg), the trachea was extubated and then the patient was transferred to the post-v care unit (PACU).

### Measurements and outcomes

The primary outcome of this study was the intraoperative anaesthetic requirement, assessed by the total consumption of fentanyl and dexmedetomidine, as well as end-tidal isoflurane concentration, with anaesthetic depth maintained within a target entropy range of 40–60. Secondary outcomes included haemodynamic stability, evaluated through continuous monitoring of heart rate and mean arterial blood pressure throughout the procedure; the quality of the surgical field, assessed using the Fromme–Boezaart scale, time to extubation and recovery pattern and any side effects or complications, such as hypotension, bradycardia, or block-related adverse events.

### Statistical analysis

Sample size calculation was performed using the NCSS 2004 and PASS 2000 software. To determine the minimum required sample size, we assumed a medium effect size (W = 0.40) for the Chi-square test of association with 1 degree of freedom, a two-sided significance level of 0.05, and a power of 80%. Under these assumptions, the calculated minimum sample size required to detect a statistically significant difference between the two groups was 60 participants. Data were entered into the computer and subjected to data analysis. Data analysis was conducted using IBM SPSS Statistics version 26. Continuous variables were tested for normality using the Shapiro-Wilk test and were presented as mean ± standard deviation (SD) or median with interquartile range (IQR), as appropriate. Categorical variables were expressed as frequencies and percentages. For between-group comparisons, continuous variables with normal distribution were analyzed using the independent samples t-test, whereas non-normally distributed data were compared using the Mann–Whitney U test. Categorical variables were evaluated using the Chi-square test or Fisher’s exact test, depending on the expected cell frequencies. Haemodynamic parameters such as heart rate and mean arterial blood pressure, recorded at multiple intraoperative time points, were analyzed using repeated-measures ANOVA to assess changes over time within groups. For ordinal data such as the Fromme–Boezaart surgical field grading, within-group comparisons across time points were conducted using the Friedman test, while between-group comparisons at each time point were performed using the Mann–Whitney U test. A p-value < 0.05 was considered statistically significant.

## Results

Among 78 patients evaluated for eligibility for endoscopic trans sphenoidal pituitary surgery (ETS), 60 fulfilled the inclusion criteria and were randomly assigned into two equal groups: the trans nasal group (Group TN, *n* = 30) and the percutaneous group (Group PC, *n* = 30) (Fig. 1).

There were no statistically significant differences between the two groups in terms of age, sex, height, weight, body mass index (BMI), or ASA physical status classification (Table 1). This indicates an appropriate baseline comparability of both study groups.

**Table 1:** Comparison between the two studied groups according to demographic data

	**Group TN(n = 30)**	**Group PC(n = 30)**	**Test of Sig.**	**p**
Age (years)	37.20 ± 11.93	42.23 ± 11.50	t=1.664	0.102
Sex				
Male	15 (50.0%)	22 (73.3%)	^χ2^=3.455	0.063
Female	15 (50.0%)	8 (26.7%)		
Height (m) Mean ± SD	1.67 ± 0.10	1.71 ± 0.10	t=1.392	0.169
Weight (kg) Mean ± SD	73.33 ± 12.90	78.73 ± 8.96	t=1.883	0.065
BMI (kg/m^2^) Mean ± SD	26.10 ± 3.47	27.22 ± 4.37	t=1.105	0.274
ASA				
I	21 (70.0%)	19 (63.3%)	^χ2^=0.300	0.584
II	9 (30.0%)	11 (36.7%)		

*SD* Standard deviation, *t* Student t-test, χ^2^ Chi square test; *p* p value for comparing between the two studied groups

*: Statistically significant at *p* ≤ 0.05

**Fig. 2:**
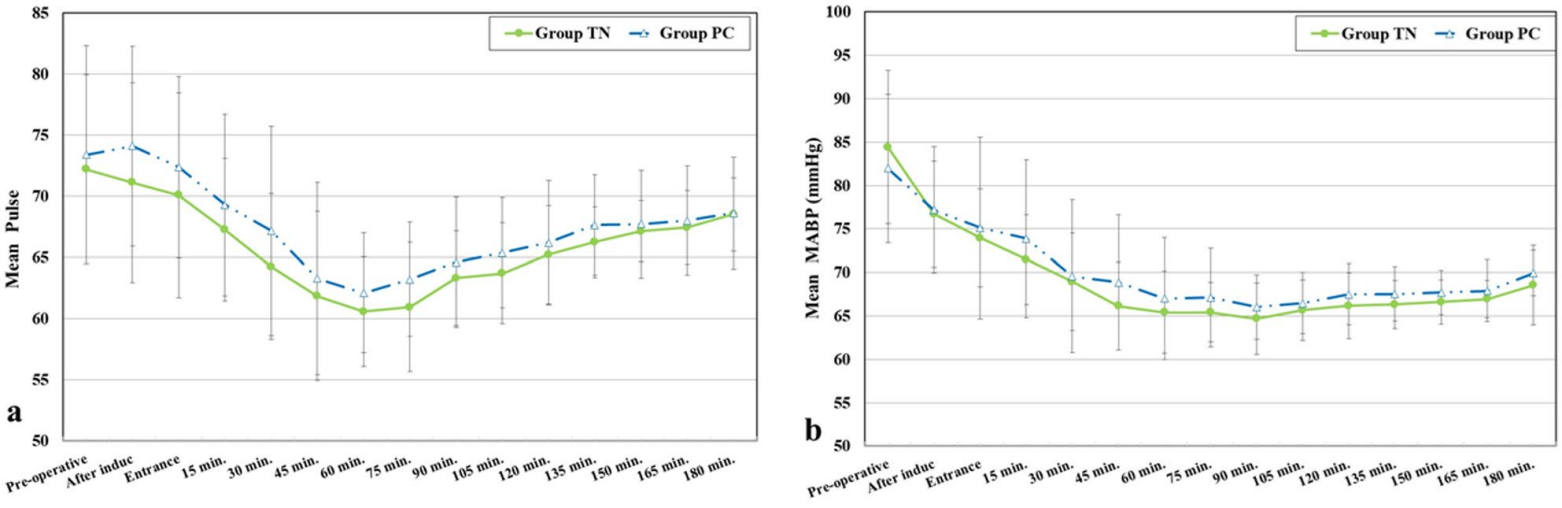
**a** Comparison between the two studied groups according to Pulse. **b** Comparison between the two studied groups according to Mean arterial blood pressure (mmHg)

No statistically significant differences were found in mean arterial blood pressure (MAP) or heart rate (HR) between the groups at any recorded intraoperative time point. The p-values for MAP ranged from 0.100 to 0.774, and for HR from 0.084 to 0.944 (Fig. 2).

Total intraoperative isoflurane consumption was significantly higher in Group TN (40.60 ± 4.71 ml) compared to Group PC (20.33 ± 6.10 ml), with a p-value < 0.001. Similarly, the mean isoflurane consumption per minute was also significantly greater in Group TN (0.24 ± 0.03 ml/min) than in Group PC (0.12 ± 0.04 ml/min) (*p* < 0.001) (Table 2). The need for intraoperative fentanyl was significantly greater in Group TN (76.7%) compared to Group PC (6.7%) (*p* < 0.001), with a standard dose of 5.0 µg administered in Group TN. Likewise, dexmedetomidine use was significantly higher in Group TN (26.7%) versus Group PC (6.7%) (*p* = 0.038). Although the mean doses of dexmedetomidine did not differ significantly between groups (*p* = 0.313).

**Table 2:** Comparison between the two studied groups according to to volume of anaesthetic agent (ml/min)

	**Group TN(n = 30)**	**Group PC(n = 30)**	**Test of Sig.**	**p**
**Fentanyl (mic) n (%)**	23(76.7%)	6 (20.0%)	^χ2^= 19.288^*^	<0.001^*^
Dose	5.0 ± 0.0	–	–	–
**Dexmedetomidine (mic) n (%)**	8(26.7%)	2(6.7%)	^χ2^=4.320^*^	0.038^*^
Dose	52.0 ± 21.83	43.50 ± 2.12	t=1.081	0.313
**Total isoflurane** **Mean ± SD**	40.60 ± 4.71	20.33 ± 6.10	t= 14.406^*^	<0.001^*^
**Total isoflurane/ min** **Mean ± SD**	0.24 ± 0.03	0.12 ± 0.04	t= 14.730^*^	<0.001^*^

*SD* Standard deviation, *t* Student t-test, χ^2^ Chi square test, *p* p value for comparing between the two studied groups

*: Statistically significant at *p* ≤ 0.05

Group TN consistently exhibited higher end-tidal isoflurane concentrations than Group PC at all measured time intervals from 15 to 180 min post-induction. Median values remained steady at 1.20% in Group TN versus 0.80% in Group PC, with all differences reaching statistical significance (*p* < 0.001) (Table 3).

**Table 3:** Comparison between the two studied groups according to End tidal isoflurane

**End tidal isoflurane**	**Group TN (n = 30)**	p_0_	**Group PC (n = 30)**	p_0_	**U**	**p**
	**Median (IQR)**		**Median (IQR)**			
**15 min.**	**1.20 (1.20 – 1.30)**		**1.0 (1.0 – 1.0)**		35.00^*^	<0.001^*^
**30 min.**	1.20 (1.10 – 1.30)	>0.05	0.80 (0.80 – 1.0)	0.005^*^	0.000^*^	<0.001^*^
**45 min.**	1.20 (1.10 – 1.20)	>0.05	0.80 (0.80 – 0.80)	<0.001^*^	0.000^*^	<0.001^*^
**60 min.**	1.20 (1.10 – 1.20)	>0.05	0.80 (0.80 – 0.80)	<0.001^*^	0.000^*^	<0.001^*^
**75 min.**	1.20 (1.10 – 1.20)	>0.05	0.80 (0.70 – 0.80)	<0.001^*^	0.000^*^	<0.001^*^
**90 min.**	1.20 (1.10 – 1.20)	>0.05	0.80 (0.70 – 0.80)	<0.001^*^	0.000^*^	<0.001^*^
**105 min.**	1.20 (1.20 – 1.20)	>0.05	0.80 (0.70 – 0.80)	<0.001^*^	0.000^*^	<0.001^*^
**120 min.**	1.20 (1.15 – 1.20)	>0.05	0.80 (0.70 – 0.80)	<0.001^*^	0.000^*^	<0.001^*^
**135 min.**	1.20 (1.20 – 1.20)	>0.05	0.80 (0.70 – 0.80)	<0.001^*^	0.000^*^	<0.001^*^
**150 min.**	1.20 (1.10 – 1.20)	>0.05	0.80 (0.70 – 0.80)	<0.001^*^	0.000^*^	<0.001^*^
**165 min.**	1.20 (1.10 – 1.20)	>0.05	0.80 (0.70 – 0.80)	<0.001^*^	0.000^*^	<0.001^*^
**180 min.**	1.20 (1.10 – 1.20)	>0.05	0.80 (0.70 – 0.80)	<0.001^*^	0.000^*^	<0.001^*^

*IQR* Inter quartile range, *U* Mann Whitney test, p_0_ p value for Friedman test for comparing between pre-operative and each period, *p* p value for comparing between the two studied groups

*: Statistically significant at *p* ≤ 0.05

Regarding Boezaart surgical field grading, both Group TN and Group PC demonstrated an initial median score of 3 (IQR: 3–4) at 15 min, indicating moderate visibility in the surgical field. Over time, both groups showed a statistically significant improvement in surgical field quality, with the median score decreasing to 2 (IQR: 2–3 or 2–2) from 30 min onward (*p* < 0.001) (Table 4). Despite the significant improvement within each group regarding the surgical field grading, there was no statistically significant when comparing the two groups at any time point (*p* = 1.000. Extubation time followed the same pattern, with Group PC demonstrating a markedly faster emergence (5.53 ± 1.9 min) than Group TN (9.90 ± 1.6 min) (*p* < 0.001).). Recovery time was significantly shorter in Group PC (9.43 ± 1.6 min) compared with Group TN (11.20 ± 1.9 min) (*p* = 0.001) with smooth pattern.

**Table 4:** Comparison between the two studied groups according to Boezaat surgical filed grading

**Boezaat surgical filed grading**	**Group TN(n = 30)**	p_0_	**Group PC(n = 30)**	p_0_	**U**	**p**
	**Median (IQR)**		**Median (IQR)**			
**15 min.**	3 (3 – 4)		3 (3 – 4)		450.0	1.000
**30 min.**	2 (2 – 3)	<0.001^*^	2 (2 – 3)	<0.001^*^	450.0	1.000
**45 min.**	2 (2 – 2)	<0.001^*^	2 (2 – 2)	<0.001^*^	450.0	1.000
**60 min.**	2 (2 – 2)	<0.001^*^	2 (2 – 2)	<0.001^*^	450.0	1.000
**75 min.**	2 (2 – 2)	<0.001^*^	2 (2 – 2)	<0.001^*^	450.0	1.000
**90 min.**	2 (2 – 2)	<0.001^*^	2 (2 – 2)	<0.001^*^	450.0	1.000
**105 min.**	2 (2 – 2)	<0.001^*^	2 (2 – 2)	<0.001^*^	450.0	1.000
**120 min.**	2 (2 – 2)	<0.001^*^	2 (2 – 2)	<0.001^*^	450.0	1.000
**135 min.**	2 (2 – 2)	<0.001^*^	2 (2 – 2)	<0.001^*^	450.0	1.000
**150 min.**	2 (2 – 2)	<0.001^*^	2 (2 – 2)	<0.001^*^	420.0	1.000
**165 min.**	2 (2 – 2)	<0.001^*^	2 (2 – 2)	<0.001^*^	420.0	1.000
**180 min.**	2 (2 – 2)	0.001^*^	2 (2 – 2)	0.001^*^	91.00	1.000

*IQR* Inter quartile range, *U* Mann Whitney test, p_0_ p value for Friedman test for comparing between 15 min. and each period, *p* p value for comparing between the two studied groups

*: Statistically significant at *p* ≤ 0.05

## Discussion

The integration of regional anaesthesia, particularly the pterygopalatine fossa block (PPFB), with general anaesthesia in endoscopic trans-sphenoidal pituitary surgery has shown promise in enhancing haemodynamic stability, reducing anaesthetic and analgesic needs, and optimizing surgical conditions by targeting sensory innervation of the nasal and sphenoidal regions [12, 13]. This study compared two PPFB techniques: the traditional transnasal (TN) endoscopic-guided approach and the ultrasound-guided percutaneous (PC) method. The aim was to assess their relative efficacy in minimizing intraoperative anaesthetic consumption, enhancing haemodynamic control, improving surgical field visibility, and reducing postoperative opioid use.

The obtained results showed that the demographic parameters (age, sex, height, weight, BMI, ASA status) were statistically comparable between the TN and PC groups. This balanced distribution confirms that proper randomization procedures were followed and strengthens the study’s internal validity by minimizing potential confounding variables.

Pulse rate demonstrated a significant decline over time in both study groups, with no statistically significant difference between them, suggesting that PPF blockade, irrespective of the technique employed, exerts a consistent sympatholytic effect throughout the intraoperative period. These findings are consistent with those of Lasheen et al., [14] who reported improved haemodynamic control and better surgical field conditions when employing endoscopic PPF blocks during functional endoscopic sinus surgery, and with Ali et al., [15] who similarly observed that PPF blockade led to a reduction in heart rate and decreased reliance on beta-blockers, emphasizing the role of parasympathetic modulation in mitigating sympathetic responses. Likewise, mean arterial pressure (MAP) exhibited a significant intraoperative decline in both groups without significant intergroup variation, a haemodynamic pattern that mirrors the results of Chaudhary et al., [16] who demonstrated minimal fluctuations in heart rate and blood pressure in a high-risk cardiac patient undergoing trans nasal pituitary surgery following ultrasound-guided bilateral PPF blockade, attributing such stability to the suppression of sympathetic outflow. This supports the notion of PPF blockade as an effective anaesthetic adjunct in attenuating pressor responses and surgical stress during highly stimulating phases of endonasal procedures. These findings are further supported by Mostafa et al., [17] who reported decreased intraoperative blood pressure variability with endoscopic-guided SPGB, thus reinforcing the evidence of its haemodynamic benefits.

The ultrasound-guided PPFB significantly decreased isoflurane inhalational requirements. End-tidal isoflurane concentrations in the PC group averaged less than half those in the TN group at every measurement point (*p* < 0.001). This aligns with Saad et al.’s report of total isoflurane consumption of 19.6 mL in the PPF-block group versus 45.7 mL in the control group (*p* < 0.001) [18]. The pronounced decrease in volatile agent use not only reflects enhanced regional analgesia but also carries important clinical implications—lower inhalational doses may reduce anaesthetic-related side effects, facilitate more rapid emergence, and shorten PACU stays. Thus, our study reinforces the utility of the ultrasound-guided percutaneous PPFB as a potent anaesthetic-sparing adjunct in endoscopic trans-sphenoidal pituitary surgery.

However, the obtained results contrast with the study by Ali et al., [15] where TN PPFB significantly reduced the need for sevoflurane to maintain a BIS value of 40–50, and Higashizawa and Koga [19] who reported decreased isoflurane consumption following infraorbital nerve block during sinonasal surgery​.

One possible explanation is that the higher end-tidal isoflurane values in the TN group might reflect compensatory anaesthetic deepening in response to intraoperative stimuli or individual variability in isoflurane pharmacodynamics. Alternatively, although the TN block may offer comparable haemodynamic stability, its local anaesthetic spread might not affect all the nociceptive pathways as effectively as the percutaneous route in some patients.

A particularly significant finding was the consistently higher adjuvant medications (fentanyl and dexmedetomidine) observed in the TN group compared to the PC group.

(*p* < 0.001 at all-time points). Given the anticipated anaesthetic-sparing effects of effective regional blockade, this result necessitates closer analysis.

This interpretation is supported by the significantly greater total anaesthetic and intraoperative fentanyl consumption in the TN group, with 76.7% requiring fentanyl versus 0% in the PC group (*p* < 0.001). Cometa et al., [20] highlighted that the trans nasal route for PPFB may yield inconsistent results due to anatomical barriers that limit effective anaesthetic diffusion, such as the nasal mucosa, the sphenopalatine foramen, and surrounding fat within the pterygopalatine fossa. In contrast, the ultrasound-guided percutaneous approach ensures direct delivery of local anaesthetic into the pterygopalatine fossa, producing more consistent and potent parasympathetic blockade.

Moreover, Tolba et al. [21] reached similar conclusions, emphasizing the superior precision and reliability of needle-based percutaneous techniques (such as infrazygomatic and suprazygomatic) over diffusion-dependent intranasal methods. Consistent with this anatomical and technical rationale, our findings demonstrate that the PC approach yielded lower anaesthetic and opioid requirements despite equivalent haemodynamic stability and surgical field conditions.

The analgesic-sparing effect of ultrasound-guided PPFB parallels the findings of Saad et al., [18] who observed that bilateral PPFB during FESS reduced intraoperative fentanyl and propranolol requirements to nearly zero and eliminated the need for nitroglycerine, compared with controls receiving general anaesthesia alone.

Abdelghafar et al. [22] approached the ultrasound-guided PPFB. Eligible patients were those diagnosed with maxillofacial cancer. Compared to the control group, the interventional group required lower concentrations of inhalational anaesthetics and reduced doses of pharmacological agents, including opioids, propranolol, and nitroglycerin, for haemodynamic control (heart rate and mean arterial blood pressure). Additionally, this group demonstrated faster emergence from anaesthesia and earlier extubation.

Regarding surgical field quality, assessed using the Boezaart score, both groups achieved similarly favourable results (median score = 2), with no statistically significant differences throughout the procedure. This outcome suggests that both techniques adequately suppressed the physiological stress responses responsible for mucosal bleeding and vascular engorgement, ensuring satisfactory endoscopic visibility. These findings are consistent with those of Saad et al., [18] who reported improved surgical field conditions following ultrasound-guided PPF block during endoscopic sinus surgery.

This study limitations include inconsistencies in the objective assessment of the operative field among the surgeons performing the procedures, as well as the use of a fixed dose of local anaesthetic; we were unable to adjust or weight the dose to achieve the optimal effect.

## Conclusions

This study reinforces the value of pterygopalatine fossa block as an effective anaesthetic adjunct during endoscopic transsphenoidal surgery. While both transnasal and ultrasound-guided percutaneous approaches confer haemodynamic stability and satisfactory surgical field conditions, the PC technique demonstrates superior anaesthetic efficiency, reflected by lower isoflurane and opioid requirements. This superiority likely stems from more accurate and consistent local anaesthetic delivery enabled by real-time ultrasound guidance. Although the TN approach remains a viable option in settings lacking ultrasound capabilities, the percutaneous route should be considered the preferred technique when resources and expertise are available.

## Supplementary Information

Supplementary Material 1.

## Data Availability

The datasets generated and analyzed during the current study are available from the corresponding author upon reasonable request.

## Abbreviations

ASA: American society of anesthesiologists
BIS: Bispectral index
BMI: Body mass index
EtCO₂: Endtidal carbon dioxide
ETS: Endoscopic transsphenoidal surgery
FESS: Functional endoscopic sinus surgery
GA: General anesthesia
HR: Heart rate
IQR: Interquartile range
IRB: Institutional review board
MABP: Mean arterial blood pressure
MAP: Mean arterial pressure
PACU: Postanesthesia care unit
PC: Percutaneous
PPF: Pterygopalatine fossa
PPFB: Pterygopalatine fossa block
PPG: Pterygopalatine ganglion
SD: Standard deviation
SPG: Sphenopalatine ganglion
TN: Transnasal
US: Ultrasound
